# Misleading Reporting (Spin) in Noninferiority Randomized Clinical Trials in Oncology With Statistically Not Significant Results: A Systematic Review

**DOI:** 10.1001/jamanetworkopen.2021.35765

**Published:** 2021-12-07

**Authors:** Chiyo Ito, Atsushi Hashimoto, Kohei Uemura, Koji Oba

**Affiliations:** 1 Graduate School of Interdisciplinary Information Studies, The University of Tokyo, Tokyo, Japan; 2 Clinical and Translational Research Center, Niigata University Medical and Dental Hospital, Niigata, Japan; 3 Interfaculty Initiative in Information Studies, The University of Tokyo, Tokyo, Japan; 4 Department of Biostatistics, School of Public Health, Graduate School of Medicine, The University of Tokyo, Tokyo, Japan

## Abstract

**Importance:**

Spin, the inaccurate reporting of randomized clinical trials (RCTs) with results that are not statistically significant for the primary end point, distorts interpretation of results and leads to misinterpretation. However, the prevalence of spin and related factors in noninferiority cancer RCTs remains unclear.

**Objective:**

To examine misleading reporting, or spin, and the associated factors in noninferiority cancer RCTs through a systematic review.

**Data Sources:**

A systematic search of the PubMed database was performed for articles published between January 1, 2010, and December 31, 2019, using the Cochrane Highly Sensitive Search Strategy.

**Study Selection:**

Two investigators independently selected studies using the inclusion criteria of noninferiority parallel-group RCTs aiming to confirm effects to cancer treatments published between January 1, 2010, and December 31, 2019, reporting results that were not statistically significant for the primary end points.

**Data Extraction and Synthesis:**

Standardized data abstraction was used to extract information concerning the trial characteristics and spin based on a prespecified definition. The main investigator extracted the trial characteristics while both readers independently evaluated the spin. The Preferred Reporting Items for Systematic Reviews and Meta-analyses (PRISMA) reporting guideline was followed.

**Main Outcomes and Measures:**

The main outcome was spin prevalence in any section of the report. Spin was defined as use of specific reporting strategies, from whatever motive, to highlight that the experimental treatment is beneficial, despite no statistically significant difference for the primary outcome, or to distract the reader from results that are not statistically significant. The associations (prevalence difference and odds ratios [ORs]) between spin and trial characteristics were also evaluated.

**Results:**

The analysis included 52 of 2752 reports identified in the PubMed search. Spin was identified in 39 reports (75.0%; 95% CI, 61.6%-84.9%), including the abstract (34 reports [65.4%; 95% CI, 51.1%-76.9%]) and the main text (38 reports [73.1%; 95% CI, 59.7%-83.3%]). Univariate analysis found that the spin prevalence was higher in reports with data managers (prevalence difference, 27%; 95% CI, 1.1%-50.3%), reports without funding from for-profit sources (prevalence difference, 31.2%; 95% CI, 4.8%-53.8%), and reports of novel experimental treatments (prevalence difference, 37.5%; 95% CI, 5.8%-64.7%). Multivariable analysis found that novel experimental treatment (OR, 4.64; 95% CI, 0.98-22.02) and funding only from nonprofit sources only (OR, 5.20; 95% CI, 1.21-22.29) were associated with spin.

**Conclusions and Relevance:**

In this systematic review, most noninferiority RCTs reporting results that were not statistically significant for the primary end points showed distorted interpretation and inaccurate reporting. The novelty of an experimental treatment and funding only from nonprofit sources were associated with spin.

## Key Points

**Question:**

Is the interpretation and reporting of noninferiority trials with primary end point results that are not statistically significant correct, and what are the associated factors of misleading reporting?

**Findings:**

This systematic review of 52 noninferiority randomized clinical trials of cancer treatments with results for primary end points that are not statistically significant, 75% included misleading reporting. Multivariable analysis found that the prevalence of misleading reporting was significantly lower in reports with funding from for-profit sources and higher in reports of novel experimental treatments.

**Meaning:**

These findings suggest that authors should carefully consider noninferiority cancer clinical trial result interpretation and reporting, especially for primary outcome results that are not statistically significant.

## Teaser

This systematic review examines misleading reporting and associated factors in noninferiority randomized clinical trials with primary results that are not statistically significant.

## Introduction

Randomized clinical trials (RCTs) are the criterion standard in research for hypothesis-based treatment efficacy and safety evaluation.^1,2^ RCTs must be performed according to predefined study protocols and statistical analysis plans.^3^ RCT results are typically interpreted based on the statistical significance of the primary end point analysis results. Trials with statistically significant results for the primary end point are known as *positive* trials, while those with results that are not significant results are *negative* trials. Both results are equally important for scientific progress if quality RCTs are planned, conducted, analyzed, and reported.^4^

However, problems can arise when reports mislead readers by distorting result interpretation and suggesting that positive results have been obtained, even if statistically significant differences have not been determined for the primary end point. This problem of misleading reporting is called *spin*.^5,6,7,8,9,10^ Boutron et al^6^ observed that RCT reports with spin were more likely to emphasize the benefit of the experimental treatment by focusing on statistically significant results, including those of secondary end points and subgroup analyses, which should be interpreted as exploratory results. Systematic reviews have previously classified spin in the field of oncology.^7,8,9,10,11^ Studies on the impact of spin have concluded that it might lead readers to overestimate result positivity.^12,13,14^

Spin in RCTs have mainly been discussed in superiority trials, with few reports assessing spin in noninferiority trials.^15^ Compared with superiority RCTs, noninferiority RCTs may have more factors that complicate interpretation, including noninferiority margin, assay sensitivity, and choice of analysis population.^16^ However, no systematic review has investigated spin prevalence in noninferiority RCTs in oncology. Given the complexities of interpretation inherent to noninferiority RCTs, we consider it is important to clarify how much spin exists and the factors associated with the spin when it is present.

Therefore, we performed a systematic review of the spin prevalence in negative noninferiority cancer RCTs published since 2010, when the US Food and Drug Administration guidelines for noninferiority trials were published.^16^ This systematic review of noninferiority RCTs investigated spin prevalence and its associated factors in oncology.

## Methods

This systematic review follows the Preferred Reporting Items for Systematic Reviews and Meta-analyses (PRISMA) reporting guideline. Data were analyzed from March 22 to September 15, 2021.

### Selection of Eligible Reports

**Figure 1.**
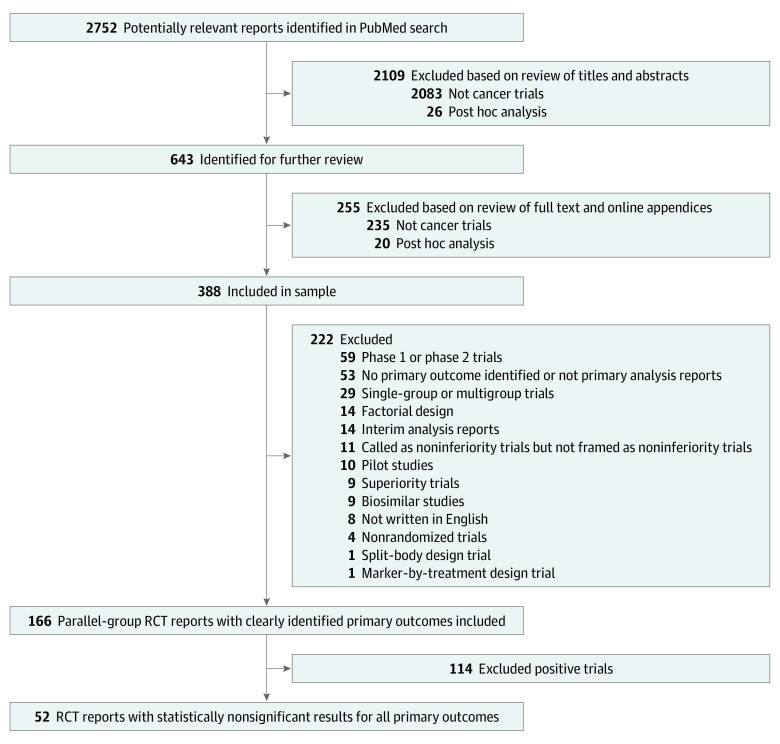
Study Selection Flowchart RCT indicates randomized clinical trial.

The selection criteria were reports on an RCT, examining anticancer treatment, using a noninferiority design, and published between January 1, 2010, and December 31, 2019. Using PubMed, we created a search strategy reflecting the inclusion criteria and systematically collected reports. This search strategy is described in the eMethods in the Supplement. The Cochrane Highly Sensitive Search Strategy^17^ was used to identify the RCTs. After collecting reports identified by the search strategy, we read the abstract and main text of each report, and excluded reports based on our exclusion criteria, including reports other than direct anticancer efficacy assessments (eg, quality of life, cost-effectiveness, diagnosis method, and primary end point could not be identified); exploratory studies (eg, phase 1, phase 2) or pilot studies; superiority or equivalent studies; other study designs (eg, crossover study, multigroup or single-group study, and factorial design); studies that terminated early at an interim analysis; and reports not written in English. We defined negative trials as those that did not show that the effect of the experimental treatment, compared with the control treatment, was better than a prespecified noninferiority margin and included them in analysis (Figure 1).

To ensure the strategy of literature collection, we confirmed that the same reports were selected by 2 authors (C.I. and A.H.), independently conducting the search. The inclusion of reports not selected by both authors was discussed among all authors.

### Extraction of Information From Selected Reports

From the selected reports, we extracted journal information (name, impact factor in 2018), general report information (publication year, number of citations in March 2021), author information (numbers of statisticians and other roles; eg, project and data managers), funding source (for-profit [eg, pharmaceutical or medical device industry], nonprofit [eg, public funds], both, no funding), rationale for conducting the study (novelty [ie, not used in clinical practice], safety, experimental treatment application simplicity), primary end point (efficacy measurement), noninferiority margin, number of patients or participants (planned and actual numbers of individuals enrolled), and analysis population. This information was recorded in a data collection form that was prepared in advance.

### Spin and Evaluation Method Definition

We defined *spin* as the “use of specific reporting strategies, from whatever motive, to highlight that the experimental treatment is beneficial, despite a statistically nonsignificant difference for the primary endpoint, or to distract the reader from statistically nonsignificant results,” as proposed by Boutron et al.^6^ We used this definition to evaluate the presence of spin in the results and conclusion sections in the abstracts, as well as the results, discussion, and conclusion sections in the main text of each report. The spin strategies were identified according to the type of research,^18,19,20,21^ and we identified spin strategies for noninferiority RCTs. Spin was considered to be present if the report corresponded to the strategies of spin specified in advance and improperly emphasized the benefit of the experimental treatment.

The 8 spin strategies specified in advance claimed the benefit of the experimental treatment (1) by emphasizing trends in point estimates, despite lacking significance for the primary end point results, denoted as *trend for primary end point*; (2) based on the results of the secondary end point, denoted as *secondary end point*; (3) based on the results of subgroup analysis, denoted as *subgroup analysis*; (4) based on the secondary analysis results of the primary end point, such as by changing the analysis population or measuring the treatment effect, denoted as *secondary analysis of the primary end point*; (5) based on the intragroup comparison results, such as those before and after treatment, denoted as *within-group comparisons*; (6) with no mention in the discussion of the experimental treatment’s unclear safety profile in reports that stated the rationale for conducting the study was safety of the experimental treatment, denoted as *no mention of safety profile*; (7) based on safety alone, despite insignificant results of the primary end point analysis in the conclusion section, denoted as *safety*; or, (8) for any other situations deemed as spin by the reviewers, denoted as *other*. No report was found in the within-group comparison category, so the category is not presented in the results.

### Spin Levels in the Conclusion Sections

The spin levels in the conclusions of the abstract and the main text were evaluated as none, low, moderate, and high. Low indicates that the report contained spin, despite mentioning that noninferiority was not confirmed; alternatively, the report did not mention that noninferiority was not confirmed but rather mentioned uncertainty in claiming the treatment benefit, as well as the need to perform additional confirmatory studies. Moderate indicates that the report did not mention that noninferiority was not confirmed and failed to mention either the presence of uncertainty to claim the experimental treatment benefit or the need for additional confirmatory studies. High indicates that the report did not mention noninferiority was not confirmed and recommended the clinical application of the experimental treatment, despite not mentioning the uncertainty in claiming the experimental treatment benefit and not mentioning the need for additional confirmatory studies.

Spin was evaluated independently by the main reviewer (C.I.) and 2 secondary reviewers (K.U. and K.O.). Disagreements were discussed to reach a final evaluation.

### End Points

The primary end point of this study was the spin prevalence in any section of the reports, including results and conclusion of the abstract and the results, discussion, and conclusion in the main text. The secondary end points were the spin prevalence in the abstract and main text sections and the spin level in the abstract and main text conclusion.

### Statistical Analysis

For discrete variables, we calculated the number of reports and percentages for each category. For continuous variables, we calculated the medians and IQRs. To determine the spin prevalence in any section of a report, we calculated the spin proportion and the 95% CI. To examine the associations between trial characteristics and prevalence of spin, we calculated the prevalence difference relative to the reference category, as well as the 95% CI. The Agresti-Coull^22^ or Agresti-Caffo methods^23^ were used for all 95% CI calculations. Logistic regression was performed to adjust for variables. Backward stepwise selection was used (*P* = .15 removed), and the Wald 95% CIs for odds ratios (ORs) were calculated. Journal specialty, publication year, journal impact factor, number of citations, presence of various author roles, funding from for-profit source, rationale of novelty, safety, application simplicity, primary outcome type (hazard ratio or survival proportion difference), noninferiority margin, planned sample size achievement, and analysis population were used as variables. All statistical analyses were performed using SAS statistical software version 9.4 (SAS Institute). Statistical significance was determined with 2-sided 95% CIs that do not cross 1.

## Results

### Study Characteristics of the Selected Reports

Our PubMed search identified 2752 studies, of which we selected 166 eligible parallel-group noninferiority RCTs, including 52 negative studies (Figure 1).^24,25,26,27,28,29,30,31,32,33,34,35,36,37,38,39,40,41,42,43,44,45,46,47,48,49,50,51,52,53,54,55,56,57,58,59,60,61,62,63,64,65,66,67,68,69,70,71,72,73,74,75^ Table 1 presents the selected study characteristics. The negative studies comprised 12 reports published from 2010 to 2013, 19 reports published from 2014 to 2016, and 21 reports published from 2017 to 2019. Two-thirds of the reports (34 reports [65.4%]) assessed drugs as experimental cancer treatments. More than half of the trials (29 reports [55.8%]) were funded by nonprofit sources only. No specific noninferiority margin was more frequently set as a trial design.

**Table 1.** Report Characteristics

Characteristic	Reports, No. (%) (N = 52)
Journal specialty	
Oncology	35 (67.3)
Nononcology	17 (32.7)
Publication year	
2010-2013	12 (23.1)
2014-2016	19 (36.5)
2017-2019	21 (40.1)
Journal impact factor in 2018, median (IQR)	14.2 (5.3-28.3)
Citations in March 2021, median (IQR), No.	35 (13-95.5)
Cancer type	
Breast	11 (21.2)
Blood	9 (17.3)
Colon	9 (17.3)
Lung	3 (5.8)
Liver	3 (5.8)
Prostate	2 (3.9)
Gastro	2 (3.9)
Uterine	2 (3.9)
Kidney	2 (3.9)
Other	9 (17.3)
Experimental treatment	
Drug	34 (65.4)
Surgical treatment or procedure	10 (18.4)
Radiation	4 (7.7)
Combination	3 (5.8)
Other	1 (1.9)
Comparator	
Drug	30 (57.7)
Surgical treatment or procedure	9 (17.3)
Radiation	7 (13.5)
Combination (drug/surgery/procedure/radiation)	6 (11.5)
Funding source	
None	0
For profit	11 (21.2)
Nonprofit	29 (55.8)
For profit and nonprofit	9 (17.3)
Not reported	3 (5.8)
Rationale for noninferiority trial (including duplicates)	
Novelty of experimental treatment	41 (78.9)
Safety of experimental treatment	32 (61.5)
Application simplicity of the experimental treatment	8 (15.4)
Not reported	9 (17.3)
Primary outcome	
OS	12 (23.1)
PFS	19 (36.5)
DFS	7 (13.5)
CRR	2 (3.9)
TTF	1 (1.9)
Other	11 (21.2)
Primary outcome (measure of treatment effect)	
HR	35 (67.3)
Difference in survival proportion at specific time point	14 (26.9)
Other	2 (3.9)
Not reported	1 (1.9)
Noninferiority margin, HR	
≤1.00 to <1.25	10 (28.6)
1.25 to <1.33	11 (31.4)
1.33 to <1.5	8 (22.9)
≥1.5	5 (14.3)
Not reported	1 (2.86)
Noninferiority margin (difference in survival proportion at specific time point)	
≤0% to <10%	6 (42.9)
10% to <15%	5 (35.7)
≤15%	3 (21.4)
Total sample size, No.	
<200	7 (13.5)
200-499	21 (40.4)
500-999	11 (21.2)
≥1000	13 (25.0)
Achievement of planned sample size	
Achieved	32 (61.5)
Not achieved	18 (34.6)
Not reported	2 (3.9)
Power of sample size calculation	
<80%	3 (5.8)
80% to <90%	38 (73.1)
≥90%	8 (15.4)
Not reported	3 (5.8)
Analysis population	
ITT	41 (78.8)
PPS	11 (21.2)
Not reported	0

Abbreviations: CRR, complete response rate; DFS, disease free survival; HR, hazard ratio; ITT, intention-to-treatment; OS, overall survival; PFS, progression free survival; PPS, per protocol set; TTF, time to treatment failure.

### Spin Overview and Extent

Table 2 shows the spin prevalence in the 52 negative studies, according to each abstract and main text section. A total of 39 reports (75.0%; 95% CI, 51.7%-84.9%) contained spin in at least 1 part. Regarding the abstracts, 34 reports (65.4%; 95% CI, 51.8%-76.9%) contained spin in at least 1 section, and 10 reports (19.2%) contained spin in all sections. One report (1.9%) contained spin the results only, and 23 reports (44.2%) contained spin in the conclusions only. Regarding the main text, 38 reports (73.1%; 95% CI, 59.7%-83.3%) had spin in at least 1 section, and 6 reports (11.5%) had spin in all sections. Finally, 3 reports (5.8%) contained spin in the discussion only, and 13 reports (25.0%) contained spin in the conclusions only. In both the abstract and main text, the conclusions section tended to show spin more often.

**Table 2.** Overview of the Spin Prevalence, Strategy, and Level of Spin

Measure	Reports, No. (%)	
	Abstract (n = 52)	Main text (n = 52)
**Presence of spin**		
Overall total, No. (%) [95% CI]	39 (75.0) [61.7-84.9]	
Total, No. (%) [95% CI]	34 (65.4) [51.8-76.9]	38 (73.1) [59.7-83.3]
Section with spin		
None	18 (34.6)	14 (26.9)
Result only	1 (1.9)	0 (0)
Discussion only	NA	3 (5.8)
Conclusion only	23 (44.2)	13 (25.0)
In 2 sections	NA	16 (30.8)
In all sections	10 (19.2)	6 (11.5)
**Strategy of spin**		
Results		
Any spin	11 (21.2)	12 (23.1)
Trend for primary end point	1 (2.0)	1 (1.9)
Secondary end point	7 (13.5)	4 (7.7)
Subgroup analysis	4 (7.7)	4 (7.7)
Secondary analysis of the primary end point	2 (3.9)	3 (5.8)
Within-group comparison	0	0
Other	2 (3.9)	3 (5.8)
Discussion	NA	19 (36.5)
Any spin	NA	19 (36.5)
Trend for primary end point	NA	4 (7.7)
Secondary end point	NA	6 (11.5)
Subgroup analysis	NA	3 (5.8)
Secondary analysis of the primary end point	NA	7 (13.5)
Within-group comparison	NA	0
No mention of safety profile	NA	2 (3.9)
Other	NA	1 (1.9)
Conclusions		
Any spin	33 (63.5)	35 (67.3)
With no consideration statistically nonsignificant primary outcome		
Trend for or secondary analysis of primary end point	5 (9.6)	5 (9.6)
Secondary end point or subgroup analysis	9 (17.3)	9 (17.3)
With acknowledgment statistically nonsignificant results for the primary outcome		
Trend for or secondary analysis of primary end point	4 (7.7)	6 (11.5)
Secondary end point or subgroup analysis	10 (19.2)	11 (21.2)
Other		
Safety	6 (11.5)	6 (11.5)
Other	6 (11.5)	8 (15.4)
Level of spin in the conclusion section		
None	19 (36.5)	17 (32.7)
Low	24 (46.2)	25 (48.1)
Moderate	1 (1.9)	1 (1.9)
High	8 (15.4)	9 (17.3)

Abbreviation: NA, not applicable.

### Strategies and Spin Levels

According to our spin classification system, 7 reports (13.5%) contained secondary end point spin in the results of the abstract. Nine reports (17.3%) included secondary end point or subgroup analysis spin with no consideration of a primary outcome that was not statistically significant, and 10 reports (19.2%) had secondary end point or subgroup analysis spin that acknowledged results that were not statistically significant for the primary outcome in the conclusion of the abstract. Six reports (11.5%) contained safety spin in the same section. In the main text, 6 reports (11.5%) contained secondary end points spin, and 7 reports (13.5%) had secondary analysis of primary end point spin in the discussion of the main text. Regarding the conclusion of the main text, 9 reports (17.3%) showed secondary end point or subgroup analysis spin without mentioning that results related to the primary outcome were not statistically significant, 11 reports (21.2%) had secondary end point or subgroup analysis spin that mentioned that results related to the primary outcome were not statistically significant, and 6 reports (11.5%) had safety spin.

We identified 33 reports (63.5%) with spin in the conclusion of the abstract; of these, 24 reports (46.2%) had low spin levels and 8 reports (15.4%) had high spin levels. Similarly, 35 reports (67.3%) had spin in the main text, with 25 reports (48.1%) showing low spin levels and 9 reports (17.3%) showing high spin levels. Examples of the conclusions for each spin level in the conclusion of the abstract are shown in eTable 1 in the Supplement.

### Factors Associated With Spin

**Figure 2.**
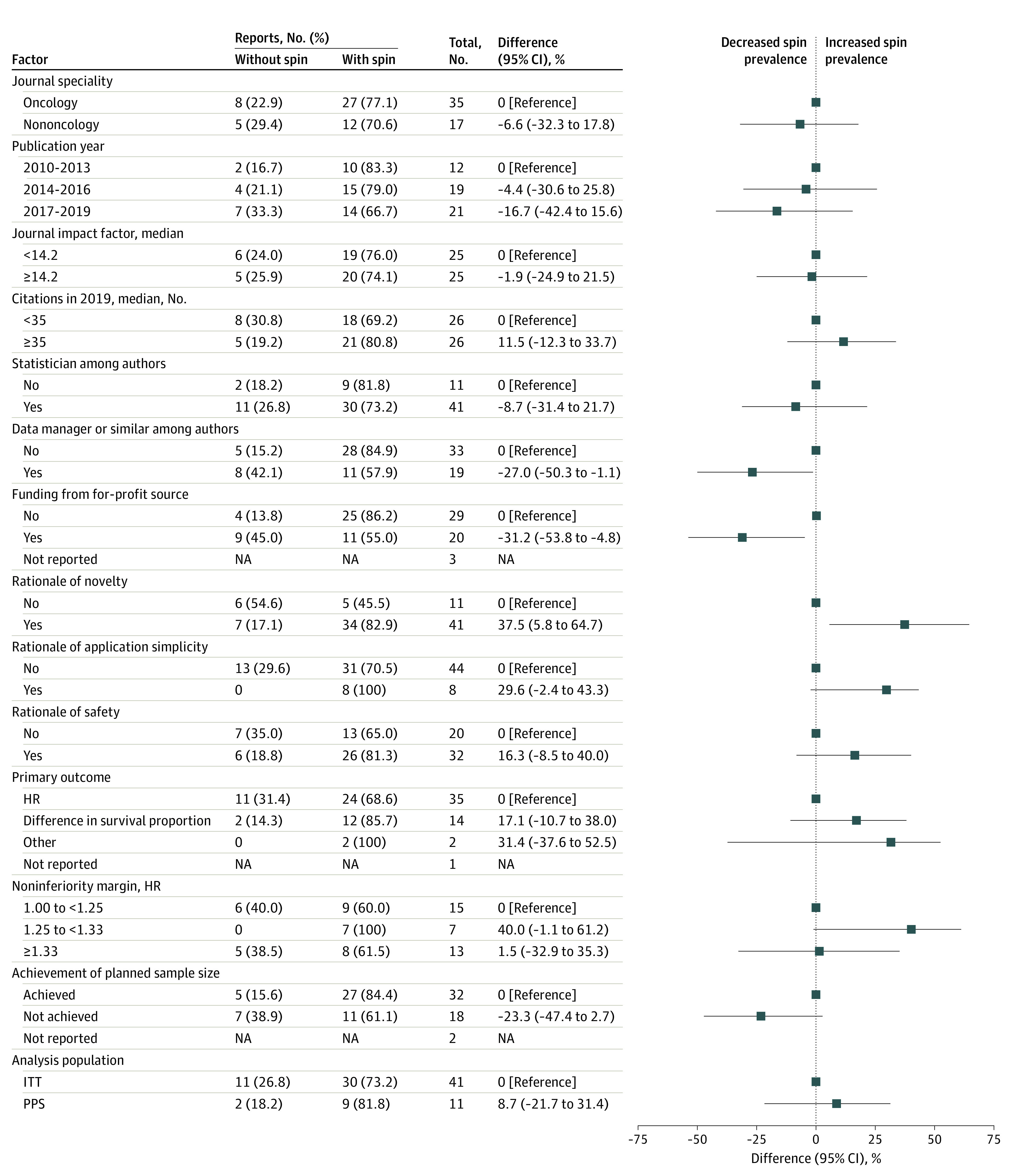
Differences in Spin Prevalence According to Different Study Characteristics HR indicates hazard ratio; ITT, intention to treat; NA, not applicable; PPS, per protocol set.

We evaluated the association between spin and the assessed variables to consider their associations with spin prevalence (Figure 2). Lack of data managers, project managers, or similar roles among authors (prevalence difference, 27.0%; 95% CI, 1.1%-50.3%) and having no funding by for-profit sources (prevalence difference, 31.2%; 95% CI, 4.8%-53.8%) were associated with higher spin prevalence. Studies with novel study treatments showed more prevalent spin (prevalence difference, 37.5%; 95% CI, 5.8%-64.7%). Considering the backward stepwise multivariable logistic regression, of 52 reports included in this study, 3 reports were excluded with no reported efficacy measurement (hazard ratio or difference in survival proportion) and funding sources, and all variables shown in Figure 2 were entered; the final model included the novelty of the study treatment and source of funding. The analysis found that reports without funding by for-profit sources (OR, 5.20; 95% CI, 1.21-22.29) and reports on novel study treatments (4.64; 95% CI, 0.98-22.02) were associated with more prevalent spin.

We examined the associations of spin level in the conclusion of the abstract for the factors with large differences in spin prevalence and relatively large numbers of studies in those categories. We did not include as targets for examination factors that had relatively few studies (eTable 2 in the Supplement).

## Discussion

**Figure 3.**
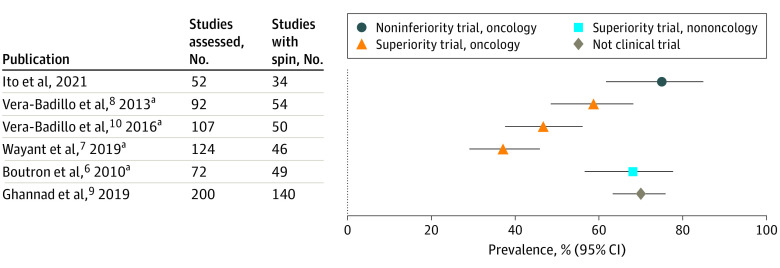
Comparison of Spin Prevalence by Clinical Study Type The x-axis shows the point estimates and 95% CIs of the prevalence of spin. ^a^Spin prevalence for the abstract only.

This systematic review found that in noninferiority oncology RCTs with results that were not statistically significant, three-quarters of reports contained spin. Direct comparisons are complicated because of the differences in study periods and the definition of spin. However, the spin prevalence was higher in these reports than in studies reporting superiority RCTs for cancer treatments^6,7,8,9,10^ (Figure 3). Certain design features in noninferiority trials make interpretation difficult, and certain regulatory authorities^16,76^ or guidelines^77^ have made recommendations for interpretation. These complexities could lead to a high spin prevalence.

We identified specific spin strategies in noninferiority RCTs. Based on our spin strategies, approximately 10% of reports focused on safety, a spin unique to noninferiority trials. Safety itself is only the rationale, or merely a prerequisite for conducting a noninferiority trial. Researchers should consider the benefit of an experimental treatment based on the results of a confirmatory analysis of efficacy, not on the safety of the treatment.

As with superiority trials, the most prevalent spin strategy was focusing on the secondary end point or subgroup analysis. This common strategy was consistent across all sections. Some spin focusing on the secondary end point or subgroup analysis was based on claiming similar efficacy without statistical consideration (eg, only with the point estimation value).

Our analysis found that the conclusions of RCTs showed the highest spin prevalence compared with other sections, a finding consistent with other reports^6,7,9^ and with important implications, as most physicians are interested only in the conclusions sections of publications.^78^ Spin-based reporting of *P* values resulted in distorted conclusions. Moreover, readers might interpret studies to have positive results even with results for the primary outcome that are not significant.^79^

Few studies have explored spin-related factors. A study by Khan et al^80^ reported that a small number of citations per year, a primary end point of efficacy or nonbinary outcome, use of a drug as a control treatment, and publication in specific journals were factors associated with high spin level in reports on superiority trials in the field of cardiology. In our study, we generated 2 hypotheses for factors associated with spin prevalence. The first factor was the rationale for the experimental treatment being novelty. To our knowledge, this is the first study to suggest the possibility of an association between the experimental treatment novelty and spin. Even if authors report negative results for an experimental treatment that is already in clinical use, a single clinical trial might not be enough to eliminate the given treatment from clinical practice. However, if the experimental treatment is not used in clinical practice, not only would the experimental treatment not become the standard of care but the results might not even get published (ie, publication bias). The second factor was a lack of funding from for-profit sources. While clinical trials funded by for-profit sources tend to report data that favor for-profit sources,^81^ and a study by Vera-Badillo et al^10^ reported that funding did not appear to be significantly associated with spin, our study found a different trend. We suggest that this could be because the organization responsible for reviewing the contents of an RCT report is likely to be well designed if the study was conducted in an environment with an external funding source. The organizational structure of an RCT is important from the perspective of the study’s feasibility and the resulting data’s reliability.^82^ Our findings suggest that the organizational structure might also be important for ensuring proper reporting. In this context, data managers might contribute not only to improving data reliability but to reducing the spin incidence in the reports.

To improve the quality of RCT reports, the International Committee of Medical Journal Editors mandated in 2004 that RCTs need to be registered for them to be published as academic reports.^83^ This requirement has resulted in an increasing proportion of cancer clinical trials registering the primary end point,^84^ as well as studies disclosing all details of the protocol in addition to the primary end point.^85^ These sustained efforts, in addition to the efforts of journal editors to reduce spin, might contribute to the decreasing trend of spin prevalence.^86^ We anticipate that more studies are likely to be reported properly in the future.

### Limitations

This study has some limitations. We only searched PubMed; therefore, this review potentially excludes studies not present in PubMed. As this review was limited to studies on cancer, the conditions of spin in reports on other diseases might differ. While we examined the associations between trial characteristics and spin prevalence, the number of reports included was too small and the characteristics were heterogenic to examine the associations between these characteristics in detail. Moreover, in the multivariable analysis, we selected variables by backward stepwise selection and the results are only useful for hypothesis generation; other factors could be confounders for the factors we found associated with spin and were impossible to adjust for.

## Conclusions

This systematic review found that 75% of negative reports on noninferiority RCTs of cancer treatments contained spin. Our results suggest that lack of funding from for-profit sources and novel experimental treatments may be associated with a high spin prevalence.

## Notes

Supplement. **eMethods.** PubMed Detailed Search Strategy **eTable 1.** Examples of Spin in the Abstract Conclusion Section **eTable 2.** Factors Associated With Level of Spin
eReferences
