# Supplementary material for: Misleading Reporting (Spin) in Noninferiority Randomized Clinical Trials in Oncology With Statistically Not Significant Results: A Systematic Review
Source: JAMA Netw Open. 2021 Dec 7;4(12):e2135765. doi: 10.1001/jamanetworkopen.2021.35765 (PMC8652604; doi:10.1001/jamanetworkopen.2021.35765)
Supplement: Supplement. — eMethods. PubMed Detailed Search Strategy eTable 1. Examples of Spin in the Abstract Conclusion Section eTable 2. Factors Associated With Level of Spin eReferences [file jamanetwopen-e2135765-s001.pdf]

Supplemental Online Content

Ito C, Hashimoto A, Uemura K, Oba K. Misleading reporting (spin) in noninferiority randomized clinical trials in oncology with statistically not significant results: a systematic review. *JAMA Netw Open*. 2021;4(12):e2135765.
doi:10.1001/jamanetworkopen.2021.35765

eMethods. PubMed Detailed Search Strategy
eTable 1. Example of Spin in the Abstract Conclusion Section
eTable 2. Factors Associated With Level of Spin
eReferences

This supplemental material has been provided by the authors to give readers additional information about their work.

© 2021 Ito C et al. *JAMA Network Open.*

eMethods. PubMed Detailed Search Strategy

(randomized controlled trial[pt] OR controlled clinical trial[pt] OR randomized[tiab] OR placebo[tiab] OR drug therapy[sh] OR randomly[tiab] OR trial[tiab] OR groups[tiab]) NOT (animals[mh] NOT human[mh])
AND
cancer[sb]
AND
(non-inferior* OR non inferior* OR noninferior* OR “not inferior*”)
AND
“2010/01/01”[Date - publication]: “2019/12/31”[Date - publication]

First search was conducted in 2019 for reports published by December 31, 2018 and second search was conducted in October 2020 for reports published by December 31, 2019.

© 2021 Ito C et al. *JAMA Network Open.*

eTable 1. Examples of Spin in the Abstract Conclusion Section

Abbreviation: PO, primary outcome; NM, non-inferiority margin; CI, confidence interval; OS, overall survival; PFS, progression-free survival; DFS: disease-free survival.

High level of spin

Article ID	Cancer type	Experimental treatment	Comparator	Result of PO (CI)	Conclusion in the abstract
1950[1] PO=OS, NM=12.75% difference in 1- year survival proportion	Advanced multiple myeloma	100mg/day of thalidomide	400 mg/day of thalidomide	7.2% (Upper limit: 15.6%)	Collectively, low-dose thalidomide 100 mg⁄day has significant activity in advanced myeloma with an improved safety profile and can be a good salvage therapy in combination with dexamethasone.
1396[2] PO= Axillary recurrence, NM=HR 2.00	Breast cancer	Axillary radiotherapy	Axillary lymph node dissection	Point estimate not reported (0.00 to 5.27)	Axillary lymph node dissection and axillary radiotherapy after a positive sentinel node provide excellent and comparable axillary control for patients with T1–2 primary breast cancer and no palpable lymphadenopathy. Axillary radiotherapy results in significantly less morbidity.
1585[3] PO=Local PFS, NM=Not reported	Glottic squamous cell carcinomas	Hypofractionation (HYPO)	Conventional fractionation (CONV)	1.551 (CI: Not reported)	Given that HYPO is at least not inferior to CONV with a similar toxicity profile, the hypofractionation scheme used in this study can be offered to patients with T1–2 glottic carcinoma with potential advantages in terms of local control and a shortened overall treatment time.

© 2021 Ito C et al. *JAMA Network Open.*

**Article ID**	**Cancer type**	**Experimental treatment**	**Comparator**	**Result of PO (CI)**	**Conclusion in the abstract**
1032[4]<br>PO=PFS,<br>NM=HR 0.82	Multiple myeloma	Melphalan, prednisone, and lenalidomide (mPR-R)	Melphalan, prednisone, and thalidomide (MPT-T)	0.84<br>(0.64 to 1.09)	In a randomized phase 3 trial, overall response rates, PFS, and overall survival were similar between MPT-T and mPR-R.<br>Toxicity with both regimens was common, but mPR-R was better tolerated, and patients on the mPR-R regimen reported better quality of life.
219[5]<br>OP=PFS,<br>NM=Not reported	Advanced or recurrent nonsquamous non-small-cell lung cancer	Pemetrexed plus bevacizumab (BevPem)	Carboplatin/ pemetrexed plus bevacizumab (BevCPem)	Not reported<br>*p*=0.7864	Results from the 65plus study give evidence that BevPem and BevCPem treatments may exert differential effects on PFS, depending on the patients ECOG PS. It appears that patients with better ECOG PS (0–1) benefited more from the combined treatment with carboplatin, while the group comprising more severely impaired patients (ECOG PS 2) benefited more from the monochemotherapy.
776[6]<br>PO=PFS,<br>NM=HR 1.3	Nonsquamous none-small-cell Lung cancer	Cisplatin with pemetrexed	Cisplatin with docetaxel	Not reported	In nonsquamous none-small-cell lung cancer patients lacking driver mutations, the PFS and response rates were similar between the 2 arms, and toxicity was tolerable, although adverse events and more severe toxicities were observed more frequently in the Doc-Cis arm.

© 2021 Ito C et al. *JAMA Network Open.*

Article ID	Cancer type	Experimental treatment	Comparator	Result of PO (CI)	Conclusion in the abstract
106[7] PO=PFS, NM=HR 1.32	Metastatic or Recurrent Gastric Cancer	Irinotecan	Paclitaxel	1.27 (0.86 to 1.88)	Although paclitaxel showed numerically longer PFS and OS compared with irinotecan, this was statistically insignificant. Both irinotecan and paclitaxel are valid second-line treatment options in MRGC.
2333[8] PO=Complete response rate, NM=10% difference	Hepato-cellular carcinoma	Percutaneous acetic acid (PAAI)	Radiofrequency ablation (RFA)	8.3% (-12.5% to 29.2%)	PAAI and RFA have similar efficacy in treating small HCC. PAAI could thus be a cost-effective alternative in situations where RFA is either unavailable or unaffordable.

Moderate level of spin

Article ID	Cancer type	Experimental treatment	Comparator	Result of PO (CI)	Conclusion in the abstract
286[9] PO=DFS, NM=-4% difference at 4-year	Pediatric Acute Lymphoblastic Leukemia	Reduced-intensity delayed intensification	Standard delayed intensification	-4% (Lower limit: -6.4%)	Although the criteria used for the standard-risk definition in this trial identified patients with exceptionally good prognosis, reduction of chemotherapy was not successful mainly because of an increased rate of relapse. The data suggest that treatment reduction is feasible in specific subgroups, which underlines the biologic heterogeneity of this cohort selected according to treatment response.

© 2021 Ito C et al. *JAMA Network Open.*

Low level of spin

Article ID	Cancer type	Experimental treatment	Comparator	Result of PO (CI)	Conclusion in the abstract
2205[10]<br>PO=Disease progression<br>NM=10% difference	Localized Prostate Cancer	Cryoablation	Radiotherapy	0.2%<br>(-10.8% to 11.2%)	The observed difference in disease progression at 36 months was small, 0.2%; however, because of the wide confidence interval, from -10.8% to 11.2%, it was not possible to rule out inferiority (defined a priori as a 10% difference). With longer term follow-up, the trend favors cryoablation. Significantly fewer positive biopsies were documented after cryoablation than after radiotherapy.
2053[11]<br>PO=OS<br>NM=HR 1.25	Solitary brain metastasis	Radiosurgery plus adjuvant whole brain radiotherapy	Surgery plus whole brain radiotherapy	0.53<br>(0.20 to 1.43)	This randomised trial encountered the accrual difficulties and consequent low statistical power commonly associated with interdisciplinary studies drawing from a small eligible population, but can contribute to future overviews on the management of solitary brain metastases.

© 2021 Ito C et al. *JAMA Network Open.*

Article ID	Cancer type	Experimental treatment	Comparator	Result of PO (CI)	Conclusion in the abstract
2101[12]<br>PO=OS<br>NM=HR 1.3	Low-Stage Renal Cell Carcinoma	Nephron-sparing surgery (NSS)	Radical nephrectomy (RN)	1.5<br>(1.03 to 2.16)	Both methods provide excellent oncologic results. In the ITT population, NSS seems to be significantly less effective than RN in terms of OS. However, in the targeted population of RCC patients, the trend in favour of RN is no longer significant. The small number of progressions and deaths from renal cancer cannot explain any possible OS differences between treatment types.
1817[13]<br>PO=PFS<br>NM=HR 1.32	Metastatic colorectal cancer	Bevacizumab alone	Bevacizumab and capecitabine plus oxaliplatin (XELOX)	1.10<br>(0.89 to 1.35)	Although the noninferiority of bevacizumab versus XELOX plus bevacizumab cannot be confirmed, we can reliably exclude a median PFS detriment >3 weeks. This study suggests that maintenance therapy with single-agent bevacizumab may be an appropriate option following induction XELOX plus bevacizumab in mCRC patients.

© 2021 Ito C et al. *JAMA Network Open.*

Article ID	Cancer type	Experimental treatment	Comparator	Result of PO (CI)	Conclusion in the abstract
1974[14]<br>PO=Recurrence-free survival<br>NM=HR 1.4	Comprehensive Surgical Staging of Uterine Cancer	Laparoscopy	Laparotomy	1.14<br>(0.92 to 1.46)	This study previously reported that laparoscopic surgical management of uterine cancer is superior for short-term safety and length-of-stay end points. The potential for increased risk of cancer recurrence with laparoscopy versus laparotomy was quantified and found to be small, providing accurate information for decision making for women with uterine cancer.
1488[15]<br>PO=OS<br>NM=HR 1.08	Unresectable, Advanced Hepatocellular Carcinoma	Brivanib	Sorafenib	1.06<br>(0.93 to 1.22)	Our study did not meet its primary end point of OS noninferiority for brivanib versus sorafenib. However, both agents had similar antitumor activity, based on secondary efficacy end points. Brivanib had an acceptable safety profile, but was less well-tolerated than sorafenib.
1715[16]<br>PO=PFS<br>NM=-0.1863 difference in the means of the logarithmic PFS times	Metastatic breast cancer	Capecitabine plus paclitaxel (XP)	Epirubicin plus paclitaxel (EP)	-0.205<br>(CI: Not reported)	Although, noninferiority of XP to EP was formally not proven, firstline XP was active and feasible. XP is a valid first-line alternative to anthracycline/taxane regimens, especially in patients previously treated with adjuvant anthracyclines.

© 2021 Ito C et al. *JAMA Network Open.*

Article ID	Cancer type	Experimental treatment	Comparator	Result of PO (CI)	Conclusion in the abstract
1748[17]<br>PO=OS<br>NM=HR 1.2	Prostate Cancer	Intermittent androgen deprivation	Continuous androgen deprivation	1.1<br>(0.99 to 1.23)	Our findings were statistically inconclusive. In patients with metastatic hormone-sensitive prostate cancer, the confidence interval for survival exceeded the upper boundary for noninferiority, suggesting that we cannot rule out a 20% greater risk of death with intermittent therapy than with continuous therapy, but too few events occurred to rule out significant inferiority of intermittent therapy. Intermittent therapy resulted in small improvements in quality of life.
1401[18]<br>PO=Local progression<br>NM=HR 1.37	Indolent lymphoma	Radiotherapy given as 4 Gy in two fractions	Radiotherapy given as a standard dose of 24 Gy in 12 fractions	3.42<br>(2.10 to 5.57)	24 Gy in 12 fractions is the more effective radiation schedule for indolent lymphoma and should be regarded as the standard of care. However, 4 Gy remains a useful alternative for palliative treatment.

© 2021 Ito C et al. *JAMA Network Open.*

Article ID	Cancer type	Experimental treatment	Comparator	Result of PO (CI)	Conclusion in the abstract
1556[19]<br>PO=Clinical success<br>NM=relative risk 0.87	Nodular and superficial basal-cell carcinoma	Imiquimod cream	Surgical excision	0.84<br>(0.78 to 0.91)	Imiquimod was inferior to surgery according to our predefined non-inferiority criterion. Although excisional surgery remains the best treatment for low-risk basal-cell carcinoma, imiquimod cream might still be a useful treatment option for small low-risk superfi cial or nodular basal-cell carcinoma dependent on factors such as patient preference, size and site of the lesion, and whether the patient has more than one lesion.
1596[20]<br>PO=OS<br>NM=20% difference at 18-month	Pediatric diffuse intrinsic pontine glioma	Hypofractionated radiotherapy	Conventional radiotherapy	2.2%<br>(-21% to 25%)	Hypofractionated radiotherapy offers lesser burden on the patients, their families and the treating departments, with nearly comparable results to conventional fractionation, though not fulfilling the non-inferiority assumption.
1043[21]<br>PO=PFS<br>NM=-7% difference at 3-year	Early-Stage Hodgkin's Lymphoma	No further treatment;	Involved-field radiotherapy	-3.8%<br>(-8.8% to 1.3%)	The results of this study did not show the noninferiority of the strategy of no further treatment after chemotherapy with regard to progression-free survival. Nevertheless, patients in this study with early-stage Hodgkin's lymphoma and negative PET findings after three cycles of ABVD had a very good prognosis either with or without consolidation radiotherapy.

© 2021 Ito C et al. *JAMA Network Open.*

Article ID	Cancer type	Experimental treatment	Comparator	Result of PO (CI)	Conclusion in the abstract
1183[22]<br>PO=complete response rate<br>NM=15% difference	Nonmuscle Invasive Bladder Cancer	Low dose bacillus Calmette-Guerin	Standard dose bacillus Calmette-Guerin	7%<br>(CI: Not reported)	The noninferiority of low dose bacillus Calmette-Guerin was not proven. However, low dose bacillus Calmette-Guerin was associated with lower toxicity and higher quality of life compared to standard dose bacillus Calmette-Guerin in patients with nonmuscle invasive bladder cancer.
1389[23]<br>PO=OS<br>NM=HR 1.0491	Advanced Hepatocellular Carcinoma	Linifanib	Sorafenib	1.046<br>(0.896 to 1.221)	Linifanib and sorafenib had similar OS in advanced HCC. Predefined superiority and noninferiority OS boundaries were not met for linifanib and the study failed to meet the primary end point. TTP and ORR favored linifanib; safety results favored sorafenib.
741[24]<br>PO=PFS<br>NM=5% difference at 3-year	Advanced Hodgkin's Lymphoma	Omit bleomycin (AVD group)	Continue ABVD (ABVD group)	1.6%<br>(-3.2% to 5.3%)	Although the results fall just short of the specified noninferiority margin, the omission of bleomycin from the ABVD regimen after negative findings on interim PET resulted in a lower incidence of pulmonary toxic effects than with continued ABVD but not significantly lower efficacy.

© 2021 Ito C et al. *JAMA Network Open.*

Article ID	Cancer type	Experimental treatment	Comparator	Result of PO (CI)	Conclusion in the abstract
894[25]<br>PO=PFS<br>NM=HR 1.388	Advanced chronic lymphocytic leukaemia	Bendamustin and rituximab	Fludarabine, cyclophosphamide, and rituximab	1.643 (1.308 to 2.064)	The combination of fludarabine, cyclophosphamide, and rituximab remains the standard front-line therapy in fit patients with chronic lymphocytic leukaemia, but bendamustine and rituximab is associated with less toxic effects.
208[26]<br>PO= Locoregional control<br>NM=15% difference	Locally Advanced Head and Neck Cancer	Cisplatin 30 mg/m^2 given once a week	Cisplatin 100 mg/m^2 given once every 3 weeks	14.6% (5.7% to 23.5%)	Once-every-3-weeks cisplatin at 100 mg/m^2 resulted in superior LRC, albeit with more toxicity, than did once-a-week cisplatin at 30 mg/m^2, and should remain the preferred chemoradiotherapy regimen for LAHNSCC in the adjuvant setting.
472[27]<br>PO=OS<br>NM=HR 1.366	Stage ll or III colon cancer	Laparoscopic D3 dissection	Open D3 dissection	1.06 (0.79 to 1.41)	Laparoscopic D3 surgery was not non-inferior to open D3 surgery in terms of overall survival for patients with stage II or III colon cancer. However, because overall survival in both groups was similar and better than expected, laparoscopic D3 surgery could be an acceptable treatment option for patients with stage II or III colon cancer.

© 2021 Ito C et al. *JAMA Network Open.*

Article ID	Cancer type	Experimental treatment	Comparator	Result of PO (CI)	Conclusion in the abstract
36[28]<br>PO=DFS<br>NM=HR 1.29	HER2-positive breast cancer	Chemotherapy plus 9 weeks trastuzumab	Chemotherapy plus 1-year trastuzumab	1.13<br>(0.89 to 1.42)	This study failed to show the non-inferiority of a shorter trastuzumab administration. One-year trastuzumab remains the standard. However, a 9-week administration decreases the risk of severe cardiac toxicity and can be an option for patients with cardiac events during treatment and for those with a low risk of relapse.
81[29]<br>PO=DFS<br>NM=HR 1.3	HER2-positive early breast cancer	Adjuvant Trastuzumab for a Duration of 9 Weeks	Adjuvant Trastuzumab for a Duration of 1 year	1.39<br>(1.12 to 1.72)	Nine weeks of trastuzumab was not noninferior to 1 year of trastuzumab when given with similar chemotherapy. Cardiac safety was better in the 9-week group. The docetaxel dosing with trastuzumab requires further study.

© 2021 Ito C et al. *JAMA Network Open.*

Article ID	Cancer type	Experimental treatment	Comparator	Result of PO (CI)	Conclusion in the abstract
247[30]<br>PO=DFS<br>NM=HR 1.12	Stage III colon cancer	Either FOLFOX (fluorouracil, leucovorin, and oxaliplatin) or CAPOX (capecitabine and oxaliplatin) administered for 3 months	Either FOLFOX (fluorouracil, leucovorin, and oxaliplatin) or CAPOX (capecitabine and oxaliplatin) administered for 6 months	1.07<br>(1.00 to 1.15)	Among patients with stage III colon cancer receiving adjuvant therapy with FOLFOX or CAPOX, noninferiority of 3 months of therapy, as compared with 6 months, was not confirmed in the overall population. However, in patients treated with CAPOX, 3 months of therapy was as effective as 6 months, particularly in the lower-risk subgroup.
250[31]<br>PO=PFS<br>NM=5% difference at 3-year	T1-2N0M0 glottic cancer	Accelerated fractionation (AF) (2.4 Gy/fraction) of 60–64.8 Gy (25–27 fractions)	Standard fractionation (SF) (2 Gy/fraction) of 66–70 Gy (33–35 fractions)	1.8%<br>(-5.1% to 8.9%)	Although the non-inferiority of AF was not confirmed statistically, the similar efficacy and toxicity of AF compared with SF, as well as the practical convenience of its fewer treatment sessions, suggest the potential of AF as a treatment option for early GC.

© 2021 Ito C et al. *JAMA Network Open.*

Article ID	Cancer type	Experimental treatment	Comparator	Result of PO (CI)	Conclusion in the abstract
465[32]<br>PO=Recurrence<br>NM=6.7% difference at 24 month	Large rectal adenomas	Endoscopic mucosal resection (EMR)	Transanal endoscopic microsurgery (TEM)	4%<br>(CI: Not reported)	Under the statistical assumptions of this study, non-inferiority of EMR could not be demonstrated. However, EMR may have potential as the primary method of choice due to a tendency of lower complication rates and a better cost-effectiveness ratio. The high rate of unexpected cancers should be dealt with in further studies.
2248[33]<br>PO=PFS<br>NM=HR 1.215	Metastatic or recurrent breast cancer	NK105	Paclitaxel (PTX)	1.255<br>(0.989 to 1.592)	The primary endpoint was not met, but NK105 had a better peripheral sensory neuropathy toxicity profile than PTX.

© 2021 Ito C et al. *JAMA Network Open.*

None

Article ID	Cancer type	Experimental treatment	Comparator	Result of PO (CI)	Conclusion in the abstract
1[34]<br>PO=Time to treatment failure<br>NM=HR 0.8	Metastatic Colorectal Cancer	Initial treatment with a fluoropyrimidine plus bevacizumab, followed by the addition of irinotecan at first progression	upfront use of fluoropyrimidine plus irinotecan plus bevacizumab	0.86<br>(0.73 to 1.02)	Noninferiority of sequential escalation therapy compared with initial combination chemotherapy could not be demonstrated for TFS. RAS status may be important to guide therapy as treatment of patients with upfront combination therapy was clearly superior in RAS/BRAF wild-type tumors, whereas sequential escalation chemotherapy seems to provide comparable results in patients with RAS mutant tumors.
32[35]<br>PO=PFS<br>NM=HR 1.34	HER2-negative metastatic breast cancer	Intermittent treatment with paclitaxel plus bevacizumab	Continuous treatment with paclitaxel plus bevacizumab	1.17<br>(0.88 to 1.57)	Intermittent first-line treatment cannot be recommended in patients with HER2-negative advanced breast cancer.

© 2021 Ito C et al. *JAMA Network Open.*

Article ID	Cancer type	Experimental treatment	Comparator	Result of PO (CI)	Conclusion in the abstract
59[36]<br>PO=OS<br>NM=HR 1.45	papillomavirus-positive oropharyngeal cancer	Radiotherapy plus cetuximab	Radiotherapy plus cisplatin	1.45<br>(Lower limit: 1.94)	For patients with HPV-positive oropharyngeal carcinoma, radiotherapy plus cetuximab showed inferior overall survival and progression-free survival compared with radiotherapy plus cisplatin. Radiotherapy plus cisplatin<br>is the standard of care for eligible patients with HPV-positive oropharyngeal carcinoma.
148[37]<br>PO=DFS<br>NM=-7.2% difference at 4.5 years	Cervical Cancer	aparoscopic or robot-assisted radical hysterectomy (minimally invasive surgery)	open abdominal radical hysterectomy (open surgery)	-10.6<br>(-16.4 to -4.7)	In this trial, minimally invasive radical hysterectomy was associated with lower rates of disease-free survival and overall survival than open abdominal radical hysterectomy among women with early-stage cervical cancer.
150[38]<br>PO=PFS<br>HR=1.225	advanced or metastatic renal cell carcinoma	sorafenib (So) followed by pazopanib (Pa)	pazopanib (Pa) followed by sorafenib (So)	1.36<br>(1.11 to 1.68)	Non-inferiority of the primary endpoint tPFS could not be demonstrated for So-Pa. The results for first-line PFS and DCR favored the Pa-So sequence.

© 2021 Ito C et al. *JAMA Network Open.*

Article ID	Cancer type	Experimental treatment	Comparator	Result of PO (CI)	Conclusion in the abstract
517[39]<br>PO=DFS<br>NM=-11% difference at 3-year	stage IIB, III colorectal cancer	Oral adjuvant uracil and protein-bound polysaccharide K (UFT/PSK)	Oral adjuvant uracil and tegafur plus leucovorin (UFT/LV)	-9.06<br>(-17.06 to -1.06)	As adjuvant chemotherapy for stage IIB and III colorectal cancer patients, UFT/PSK adjuvant therapy was not non-inferior to UFT/LV therapy with respect to the DFS.
579[40]<br>PO=relapse-free survival<br>NM=HR 1.34	Clinical Stage II/III Lower Rectal Cancer	mesorectal excision (ME) alone	mesorectal excision (ME) with lateral lymph node dissection(LLND)	1.07<br>(0.84 to 1.36)	The noninferiority of ME alone to ME with LLND was not confirmed in the intent-to-treat analysis. ME with LLND had a lower local recurrence, especially in the lateral pelvis, compared to ME alone.

© 2021 Ito C et al. *JAMA Network Open.*

Article ID	Cancer type	Experimental treatment	Comparator	Result of PO (CI)	Conclusion in the abstract
755[41]<br>PO=DFS<br>NM=1.53	HER2-negative, axillarylymph node-positive early breast cancer	epirubicin, 5-fluorouracil and cyclophosphamide every 2 weeks for four cycles, followed by four cycles of docetaxel every 2 weeks with prophylactic G-CSF support (FEC→D)	docetaxel and cyclophosphamide every 21 days for six cycles (TC)	1.147 (0.716 to 1.839)	This trial did not clearly demonstrate that TC is non-inferior to dose-dense FEC→D. However, 3-year DFS rates were excellent in both arms for women with node-positive, HER2-negative early breast cancer.

© 2021 Ito C et al. *JAMA Network Open.*

Article ID	Cancer type	Experimental treatment	Comparator	Result of PO (CI)	Conclusion in the abstract
811[42]<br>PO=PFS<br>NM=HR 1.15	Anemic patients with metastatic breast cancer	Epoetin alfa (EPO) 40,000 IU subcutaneously once a week	Best standard of care	1.089<br>(0.988 to 1.2)	The primary end point, PFS based on investigator-determined PD, did not meet noninferiority criteria. As a consistency assessment with the primary finding, PFS based on independent review committee–determined PD met noninferiority criteria. Overall, this study did not achieve noninferiority objective in ruling out a 15% increased risk in PD/death. RBC transfusion should be the preferred approach for the management of anemia in this population.
948[43]<br>PO=PFS<br>NM=HR 1.3	Previously treated advanced lung adenocarcinoma	gefitinib	erlotinib	1.125<br>(0.940 to 1.347)	The study did not demonstrate noninferiority of gefitinib compared with erlotinib in terms of PFS in patients with lung adenocarcinoma according to the predefined criteria.
1119[44]<br>PO=PFS<br>NM=1.25	Human epidermal growth factor receptor 2–positive advanced breast cancer	lapatinib and taxane therapy	tratuzumab and taxane therapy	1.37<br>(1.13 to 1.65)	As first-line therapy for HER2-positive metastatic BC, lapatinib combined with taxane was associated with shorter PFS and more toxicity compared with trastuzumab combined with taxane.

© 2021 Ito C et al. *JAMA Network Open.*

Article ID	Cancer type	Experimental treatment	Comparator	Result of PO (CI)	Conclusion in the abstract
1123[45]<br>PO=DFS<br>NM=HR 1.53	HER2-positive breast cancer	6 months of adjuvant trastumab	12 months of adjuvant trastumab	1.57<br>(0.86 to 2.1)	Our study failed to show noninferiority for the 6-month arm. The results further support the current standard of care that is administration of adjuvant trastuzumab for 12 months.
1203[46]<br>PO=OS<br>NM=HR 0.9	Primary CNS lymphoma (PCNSL)	High-dose methotrexate(HDMTX)–based chemotherapy alone	High-dose methotrexate(HDMTX)–based chemotherapy followed by whole-brain radiotherapy (WBRT)	1.03<br>(0.79 to 1.35)	Although the statistical proof of noninferiority regarding OS was not given, our results suggest no worsening of OS without WBRT in primary therapy of PCNSL.

© 2021 Ito C et al. *JAMA Network Open.*

Article ID	Cancer type	Experimental treatment	Comparator	Result of PO (CI)	Conclusion in the abstract
1225[47]<br>PO=time to progression (TTP)<br>NM=0.727	Metastatic colorectal cancer	No treatment	Continuing bevacizumab	0.74<br>(0.58 to 0.96)	Non-inferiority could not be demonstrated for treatment holidays versus continuing bevacizumab monotheray, after 4–6 months of standard first-line chemotherapy plus bevacizumab. Based on no impact on overall survival and increased treatment costs, bevacizumab as a single agent is of no meaningful therapeutic value. More efficient treatment approaches are needed to maintain control of stabilized disease following induction therapy.
1304[48]<br>PO=OS<br>NM=HR 1.50	Resectable gastric cancer	D2 gastrectomy without bursectomy	D2 gastrectomy with bursectomy	1.4<br>(0.87 to 2.25)	The final analysis could not demonstrate the noninferiority of the omission of bursectomy. Bursectomy should not be abandoned as a futile procedure.

© 2021 Ito C et al. *JAMA Network Open.*

Article ID	Cancer type	Experimental treatment	Comparator	Result of PO (CI)	Conclusion in the abstract
1576[49]<br>PO=PFS<br>NM=-10.65% difference at 12 weeks	Advanced colorectal cancer (CRC)	3-weekly irinotecan at 140 mg/m^2 (120 mg/m^2 if age >70 or PS = 2) with ciclosporin 3 mg/kg t.d.s. for three days by mouth starting on the morning before irinotecan.	Irinotecan 3-weekly 350 mg/m^2 (or 300 mg/m^2 if age >70 or performance status (PS) = 2)	-6.3<br>(-13.8 to 1.3)	The pharmacokinetic biomodulation of irinotecan using oral ciclosporin does not improve the therapeutic index of irinotecan in advanced CRC.
1627[50]<br>PO=time to progression (TTP)<br>NM=HR 1.25	Hormone-receptor-positive advanced breast cancer	Exemestane	Anastrozole	1.007<br>(0.771 to 1.317)	In this study, the efficacy and safety profiles of exemestane were similar to those of anastrozole in Japanese patients with advanced, hormone-receptor-positive breast cancer; however, TTP non-inferiority of exemestane versus anastrozole was not confirmed.

© 2021 Ito C et al. *JAMA Network Open.*

Article ID	Cancer type	Experimental treatment	Comparator	Result of PO (CI)	Conclusion in the abstract
2146[51]<br>PO=OS<br>NM=HR 0.9	Primary CNS lymphoma	First-line chemotherapy based on high-dose methotrexate without subsequent whole brain radiotherapy	First-line chemotherapy based on high-dose methotrexate with subsequent whole brain radiotherapy	1.06<br>(0.8 to 1.4)	No significant difference in overall survival was recorded when whole brain radiotherapy was omitted from first-line chemotherapy in patients with newly diagnosed primary CNS lymphoma, but our primary hypothesis was not proven. The progression-free survival benefit afforded by whole brain radiotherapy has to be weighed against the increased risk of neurotoxicity in long-term survivors.

© 2021 Ito C et al. *JAMA Network Open.*

Article ID	Cancer type	Experimental treatment	Comparator	Result of PO (CI)	Conclusion in the abstract
2436[52]<br>PO=PFS<br>NM=HR 3.01	Early-stage favorable Hodgkin lymphoma	PET-guided treatment, omitting involved-field radiotherapy after negative PET-2 (Deauville score,3)	Standard CMT(Combined-modality treatment) of 2 ×ABVD (doxorubicin, bleomycin, vinblastine, and dacarbazine) and 20-Gy involved-field radiotherapy	1.78<br>(1.02 to 3.12)	In early-stage favorable HL, a positive PET after two cycles ABVD indicates a high risk for treatment failure, particularly when a Deauville score of 4 is used as a cutoff for positivity. In PET-2–negative patients, radiotherapy cannot be omitted from CMT without clinically relevant loss of tumor control.

© 2021 Ito C et al. *JAMA Network Open.*

**eTable 2.** Factors Associated With Level of Spin					
**Factor**		**No. (%)**			
		**None**	**Low**	**Moderate**	**High**
Journal speciality	Oncology journal	13 (37.1)	16 (45.7)	1 (2.9)	5 (14.3)
	Non-oncology journal	6 (35.3)	8 (47.1)	0 (0)	3 (17.7)
Publication year	2010-2013	3 (25.0)	8 (66.7)	0 (0)	1 (8.3)
	2014-2016	8 (42.1)	8 (42.1)	0 (0)	3 (15.8)
	2017-2019	8 (38.1)	8 (38.1)	1 (4.8)	4 (19.1)
Statistician among authors	Yes	14 (34.2)	21 (51.2)	1 (2.4)	5 (12.2)
	No	5 (45.5)	3 (27.3)	0 (0)	3 (27.3)
Data manager or similar among authors	Yes	9 (47.4)	8 (42.1)	0 (0)	2 (10.5)
	No	10 (30.3)	16 (48.5)	1 (3.0)	6 (18.2)
Funding from for-profit source	Yes	10 (50.0)	7 (35.0)	0 (0)	3 (15.0)
	No	9 (31.0)	15 (51.7)	1 (3.5)	4 (13.8)
Rationale of novelty	Yes	12 (29.3)	22 (53.7)	1 (2.4)	6 (14.6)
	No	7 (63.6)	2 (18.2)	0 (0)	2 (18.2)
Rationale of application simplicity	Yes	1 (12.5)	5 (62.5)	0 (0)	2 (25.0)
	No	18 (40.9)	19 (43.2)	1 (2.3)	6 (13.6)
Rationale of safety	Yes	11 (34.4)	16 (50.0)	1 (3.1)	4 (12.5)
	No	8 (40.0)	8 (40.0)	0 (0)	4 (20.0)
Primary outcome	HR	16 (45.7)	14 (40.0)	0 (0)	5 (14.3)
	Difference in survival proportion	3 (21.4)	8 (57.1)	1 (7.1)	2 (14.3)
Non-inferiority margin (HR)	1.00≤, <1.25	7 (46.7)	6 (40.0)	0 (0)	2 (13.3)
	1.25≤, <1.33	1 (14.3)	4 (57.1)	0 (0)	2 (28.6)
	1.33≤	8 (61.5)	4 (30.1)	0 (0)	1 (7.7)
Achievement of planned sample size	Achieved	10 (31.3)	16 (50.0)	1 (3.1)	5 (15.6)
	Not achieved	8 (44.4)	7 (38.9)	0 (0)	3 (16.7)

© 2021 Ito C et al. *JAMA Network Open.*

eReferences

1. Yakoub-Agha I, Mary J-Y, Hulin C, et al. Low-dose vs. high-dose thalidomide for advanced multiple myeloma: a prospective trial from the Intergroupe Francophone du Myélome. *Eur J Haematol*. 2012;88(3):249-259.
2. Donker M, van Tienhoven G, Straver ME, et al. Radiotherapy or surgery of the axilla after a positive sentinel node in breast cancer (EORTC 10981-22023 AMAROS): a randomised, multicentre, open-label, phase 3 non-inferiority trial. *Lancet Oncol*. 2014;15(12):1303-1310.
3. Moon SH, Cho KH, Chung EJ, et al. A prospective randomized trial comparing hypofractionation with conventional fractionation radiotherapy for T1-2 glottic squamous cell carcinomas: Results of a Korean Radiation Oncology Group (KROG-0201) study. *Radiother Oncol*. 2014;110(1):98-103.
4. Stewart AK, Jacobus S, Fonseca R, et al. Melphalan, prednisone, and thalidomide vs melphalan, prednisone, and lenalidomide (Ecog E1A06) in untreated multiple myeloma. *Blood*. 2015;126(11):1294-1301.
5. Schuette W, Schneider C-P, Engel-Riedel W, et al. 65Plus: open-label study of bevacizumab in combination with pemetrexed or pemetrexed/carboplatin as first-line treatment of patients with advanced or recurrent nonsquamous non-small-cell lung cancer. *Lung Cancer (Auckl)*. 2017;8:217-229.
6. Park C-K, Oh I-J, Kim K-S, et al. Randomized phase III study of docetaxel plus cisplatin versus pemetrexed plus cisplatin as first-line treatment of nonsquamous non-small-cell lung cancer: A TRAIL trial. *Clin Lung Cancer*. 2017;18(4):e289-e296.
7. Lee K-W, Maeng CH, Kim T-Y, et al. A phase III study to compare the efficacy and safety of paclitaxel versus irinotecan in patients with metastatic or recurrent gastric cancer who failed in first-line therapy (KCSG ST10-01). *Oncologist*. 2019;24(1):18-e24.
8. Paul SB, Acharya SK, Gamanagatti SR, Sreenivas V, Shalimar S, Gulati MS. Acetic acid versus radiofrequency ablation for the treatment of hepatocellular carcinoma: A randomized controlled trial. *Diagn Interv Imaging*. 2020;101(2):101-110.
9. Schrappe M, Bleckmann K, Zimmermann M, et al. Reduced-intensity delayed intensification in standard-risk pediatric acute lymphoblastic leukemia defined by undetectable minimal residual disease: Results of an international randomized trial(AIEOP-BFM ALL 2000). *J Clin Oncol*. 2018;36(3):244-253.
10. Donnelly BJ, Saliken JC, Brasher PMA, et al. A randomized trial of external beam radiotherapy versus cryoablation in patients with localized prostate cancer. *Cancer*. 2010;116(2):323-330.
11. Roos DE, Smith JG, Stephens SW. Radiosurgery versus surgery, both with adjuvant whole brain radiotherapy, for solitary brain metastases: a randomised controlled trial. *Clin Oncol (R Coll Radiol)*. 2011;23(9):646-651.
12. Van Poppel H, Da Pozzo L, Albrecht W, et al. A prospective, randomised EORTC intergroup phase

© 2021 Ito C et al. *JAMA Network Open.*

3 study comparing the oncologic outcome of elective nephron-sparing surgery and radical nephrectomy for low-stage renal cell carcinoma. *Eur Urol*. 2011;59(4):543-552.

13. Díaz-Rubio E, Gómez-España A, Massutí B, et al. First-line XELOX plus bevacizumab followed by XELOX plus bevacizumab or single-agent bevacizumab as maintenance therapy in patients with metastatic colorectal cancer: the phase III MACRO TTD study. *Oncologist*. 2012;17(1):15-25.
14. Walker JL, Piedmonte MR, Spirtos NM, et al. Recurrence and survival after random assignment to laparoscopy versus laparotomy for comprehensive surgical staging of uterine cancer: Gynecologic Oncology Group LAP2 Study. *J Clin Oncol*. 2012;30(7):695-700.
15. Johnson PJ, Qin S, Park J-W, et al. Brivanib versus sorafenib as first-line therapy in patients with unresectable, advanced hepatocellular carcinoma: results from the randomized phase III BRISK-FL study. *J Clin Oncol*. 2013;31(28):3517-3524.
16. Lück H-J, Du Bois A, Loibl S, et al. Capecitabine plus paclitaxel versus epirubicin plus paclitaxel as first-line treatment for metastatic breast cancer: efficacy and safety results of a randomized, phase III trial by the AGO Breast Cancer Study Group. *Breast Cancer Res Treat*. 2013;139(3):779-787.
17. Hussain M, Tangen CM, Berry DL, et al. Intermittent versus continuous androgen deprivation in prostate cancer. *N Engl J Med*. 2013;368(14):1314-1325.
18. Hoskin PJ, Kirkwood AA, Popova B, et al. 4 Gy versus 24 Gy radiotherapy for patients with indolent lymphoma (Fort): a randomised phase 3 non-inferiority trial. *Lancet Oncol*. 2014;15(4):457-463.
19. Bath-Hextall F, Ozolins M, Armstrong SJ, et al. Surgical excision versus imiquimod 5% cream for nodular and superficial basal-cell carcinoma (Sins): a multicentre, non-inferiority, randomised controlled trial. *Lancet Oncol*. 2014;15(1):96-105.
20. Zaghloul MS, Eldebawy E, Ahmed S, et al. Hypofractionated conformal radiotherapy for pediatric diffuse intrinsic pontine glioma (Dipg): a randomized controlled trial. *Radiother Oncol*. 2014;111(1):35-40.
21. Radford J, Illidge T, Counsell N, et al. Results of a trial of pet-directed therapy for early-stage hodgkin's lymphoma. *N Engl J Med*. 2015;372(17):1598-1607.
22. Yokomizo A, Kanimoto Y, Okamura T, et al. Randomized controlled study of the efficacy, safety and quality of life with low dose bacillus calmette-guérin instillation therapy for nonmuscle invasive bladder cancer. *J Urol*. 2016;195(1):41-46.
23. Cainap C, Qin S, Huang W-T, et al. Linifanib versus Sorafenib in patients with advanced hepatocellular carcinoma: results of a randomized phase III trial. *J Clin Oncol*. 2015;33(2):172-179.
24. Johnson P, Federico M, Kirkwood A, et al. Adapted treatment guided by interim pet-ct scan in advanced hodgkin's lymphoma. *N Engl J Med*. 2016;374(25):2419-2429.
25. Eichhorst B, Fink A-M, Bahlo J, et al. First-line chemoimmunotherapy with bendamustine and rituximab versus fludarabine, cyclophosphamide, and rituximab in patients with advanced chronic lymphocytic leukaemia (Cll10): an international, open-label, randomised, phase 3, non-inferiority

© 2021 Ito C et al. *JAMA Network Open.*

trial. *Lancet Oncol*. 2016;17(7):928-942.

26. Noronha V, Joshi A, Patil VM, et al. Once-a-week versus once-every-3-weeks cisplatin chemoradiation for locally advanced head and neck cancer: a phase iii randomized noninferiority trial. *J Clin Oncol*. 2018;36(11):1064-1072.
27. Kitano S, Inomata M, Mizusawa J, et al. Survival outcomes following laparoscopic versus open D3 dissection for stage II or III colon cancer (Jcog0404): a phase 3, randomised controlled trial. *Lancet Gastroenterol Hepatol*. 2017;2(4):261-268.
28. Conte P, Frassoldati A, Bisagni G, et al. Nine weeks versus 1 year adjuvant trastuzumab in combination with chemotherapy: final results of the phase III randomized Short-HER study‡. *Ann Oncol*. 2018;29(12):2328-2333.
29. Joensuu H, Fraser J, Wildiers H, et al. Effect of adjuvant trastuzumab for a duration of 9 weeks vs 1 year with concomitant chemotherapy for early human epidermal growth factor receptor 2-positive breast cancer: the sold randomized clinical trial. *JAMA Oncol*. 2018;4(9):1199-1206.
30. Grothey A, Sobrero AF, Shields AF, et al. Duration of adjuvant chemotherapy for stage iii colon cancer. *N Engl J Med*. 2018;378(13):1177-1188.
31. Kodaira T, Kagami Y, Shibata T, et al. Results of a multi-institutional, randomized, non-inferiority, phase III trial of accelerated fractionation versus standard fractionation in radiation therapy for T1-2N0M0 glottic cancer: Japan Clinical Oncology Group Study (Jcog0701). *Ann Oncol*. 2018;29(4):992-997.
32. Barendse RM, Musters GD, de Graaf EJR, et al. Randomised controlled trial of transanal endoscopic microsurgery versus endoscopic mucosal resection for large rectal adenomas (Trend study). *Gut*. 2018;67(5):837-846.
33. Fujiwara Y, Mukai H, Saeki T, et al. A multi-national, randomised, open-label, parallel, phase III non-inferiority study comparing NK105 and paclitaxel in metastatic or recurrent breast cancer patients. *Br J Cancer*. 2019;120(5):475-480.
34. Modest DP, Fischer von Weikersthal L, Decker T, et al. Sequential versus combination therapy of metastatic colorectal cancer using fluoropyrimidines, irinotecan, and bevacizumab: a randomized, controlled study—xelaviri(Aio krk0110). *J Clin Oncol*. 2019;37(1):22-32.
35. Claessens AKM, Bos MEMM, Lopez-Yurda M, et al. Intermittent versus continuous first-line treatment for HER2-negative metastatic breast cancer: the Stop & Go study of the Dutch Breast Cancer Research Group (Boog). *Breast Cancer Res Treat*. 2018;172(2):413-423.
36. Gillison ML, Trotti AM, Harris J, et al. Radiotherapy plus cetuximab or cisplatin in human papillomavirus-positive oropharyngeal cancer (Nrg oncology rtog 1016): a randomised, multicentre, non-inferiority trial. *Lancet*. 2019;393(10166):40-50.
37. Ramirez PT, Frumovitz M, Pareja R, et al. Minimally invasive versus abdominal radical hysterectomy for cervical cancer. *N Engl J Med*. 2018;379(20):1895-1904.

© 2021 Ito C et al. *JAMA Network Open.*

38. Retz M, Bedke J, Bögemann M, et al. SWITCH II: Phase III randomized, sequential, open-label study to evaluate the efficacy and safety of sorafenib-pazopanib versus pazopanib-sorafenib in the treatment of advanced or metastatic renal cell carcinoma (AUO AN 33/11). *Eur J Cancer*. 2019;107:37-45.
39. Miyake Y, Nishimura J, Kato T, et al. Phase iii trial comparing uft + psk to uft + lv in stage iib, iii colorectal cancer(Mcsgo-cctg). *Surg Today*. 2018;48(1):66-72.
40. Fujita S, Mizusawa J, Kanemitsu Y, et al. Mesorectal excision with or without lateral lymph node dissection for clinical stage ii/iii lower rectal cancer (Jcog0212): a multicenter, randomized controlled, noninferiority trial. *Ann Surg*. 2017;266(2):201-207.
41. Mavroudis D, Matikas A, Malamos N, et al. Dose-dense FEC followed by docetaxel versus docetaxel plus cyclophosphamide as adjuvant chemotherapy in women with HER2-negative, axillary lymph node-positive early breast cancer: a multicenter randomized study by the Hellenic Oncology Research Group (Horg). *Ann Oncol*. 2016;27(10):1873-1878.
42. Leyland-Jones B, Bondarenko I, Nemsadze G, et al. A randomized, open-label, multicenter, phase iii study of epoetin alfa versus best standard of care in anemic patients with metastatic breast cancer receiving standard chemotherapy. *J Clin Oncol*. 2016;34(11):1197-1207.
43. Urata Y, Katakami N, Morita S, et al. Randomized phase iii study comparing gefitinib with erlotinib in patients with previously treated advanced lung adenocarcinoma: wjog 5108l. *J Clin Oncol*. 2016;34(27):3248-3257.
44. Gelmon KA, Boyle FM, Kaufman B, et al. Lapatinib or trastuzumab plus taxane therapy for human epidermal growth factor receptor 2-positive advanced breast cancer: final results of ncic ctg ma. 31. *J Clin Oncol*. 2015;33(14):1574-1583.
45. Mavroudis D, Saloustros E, Malamos N, et al. Six versus 12 months of adjuvant trastuzumab in combination with dose-dense chemotherapy for women with HER2-positive breast cancer: a multicenter randomized study by the Hellenic Oncology Research Group (Horg). *Ann Oncol*. 2015;26(7):1333-1340.
46. Korfel A, Thiel E, Martus P, et al. Randomized phase III study of whole-brain radiotherapy for primary CNS lymphoma. *Neurology*. 2015;84(12):1242-1248.
47. Koeberle D, Betticher DC, von Moos R, et al. Bevacizumab continuation versus no continuation after first-line chemotherapy plus bevacizumab in patients with metastatic colorectal cancer: a randomized phase III non-inferiority trial (SAKK 41/06). *Ann Oncol*. 2015;26(4):709-714.
48. Hirao M, Kurokawa Y, Fujita J, et al. Long-term outcomes after prophylactic bursectomy in patients with resectable gastric cancer: Final analysis of a multicenter randomized controlled trial. *Surgery*. 2015;157(6):1099-1105.
49. Middleton G, Brown S, Lowe C, et al. A randomised phase III trial of the pharmacokinetic biomodulation of irinotecan using oral ciclosporin in advanced colorectal cancer: results of the

© 2021 Ito C et al. *JAMA Network Open.*

Panitumumab, Irinotecan & Ciclosporin in COLOrectal cancer therapy trial (Piccolo). *Eur J Cancer*. 2013;49(16):3507-3516.

50. Iwata H, Masuda N, Ohno S, et al. A randomized, double-blind, controlled study of exemestane versus anastrozole for the first-line treatment of postmenopausal Japanese women with hormone-receptor-positive advanced breast cancer. *Breast Cancer Res Treat*. 2013;139(2):441-451.
51. Thiel E, Korfel A, Martus P, et al. High-dose methotrexate with or without whole brain radiotherapy for primary CNS lymphoma (G-pcnsl-sg-1): a phase 3, randomised, non-inferiority trial. *Lancet Oncol*. 2010;11(11):1036-1047.
52. Fuchs M, Goergen H, Kobe C, et al. Positron emission tomography-guided treatment in early-stage favorable hodgkin lymphoma: final results of the international, randomized phase iii hd16 trial by the german hodgkin study group. *J Clin Oncol*. 2019;37(31):2835-2845.

© 2021 Ito C et al. *JAMA Network Open.*
